# Characteristics of the immunogenicity and tumor immune microenvironment in *HER2*-amplified lung adenocarcinoma

**DOI:** 10.3389/fimmu.2022.1042072

**Published:** 2022-12-15

**Authors:** Qinyang Wang, Ziyang Mao, Wenyuan Li, Shumei Wang, Lei Wang, Lin Chen, Zhe Yang, Xiaolan Fu, Panpan Jiang, Yixue Bai, Longwen Xu, Shirong Zhang, Yuzhu Hou, Xiaohui Jia, Lili Jiang, Mengjie Liu, Guanjun Zhang, Yina Jiang, Hui Guo

**Affiliations:** ^1^ Department of Medical Oncology, The First Affiliated Hospital of Xi’an Jiaotong University, Xi’an, China; ^2^ Department of Pathology, Tangdu Hospital, The Second Affiliated Hospital of Air Force Military Medical University, Xi’an, China; ^3^ Department of Thoracic Surgery, Tangdu Hospital, The Second Affiliated Hospital of Air Force Military Medical University, Xi’an, China; ^4^ Department of Pathology, Shaanxi Provincial People’s Hospital, Xi’an, China; ^5^ Department of Pathology, The First Affiliated Hospital of Xi’an Jiaotong University, Xi’an, China; ^6^ Department of Pathogenic Microbiology and Immunology, School of Basic Medical Sciences, Xi’an Jiaotong University, Xi’an, China; ^7^ Centre for Translational Medicine, The First Affiliated Hospital of Xi’an Jiaotong University, Xi’an, China; ^8^ Key Laboratory of Environment and Genes Related to Diseases, Xi’an Jiaotong University, Ministry of Education of China, Xi’an, China

**Keywords:** HER2, amplification, lung adenocarcinoma, immunogenicity, tumor immune microenvironment

## Abstract

**Objective:**

Besides breast and gastric cancer, *HER2* amplification/mutation are also found in lung adenocarcinoma (LUAD). However, the correlation between *HER2* variations and the phenotype of immunogenicity and tumor immune microenvironment (TIME) in LUAD compared with breast and gastric cancer has yet to be fully elucidated.

**Methods:**

We integrated public databases (discovery set) and internal data (validated set) of 288 patients representing three distinct *HER2*-altered tumors. Genomic data were used to identify somatic mutations, copy number variations, and calculate tumor mutational burden (TMB) and microsatellite instability score. RNA sequencing was conducted to estimate immune gene signatures and contents of tumor-infiltrating immune cell populations. Finally, IHC was used to determine PD-L1 expression and the tumoral-infiltration of immune cells in 50 *HER2*-variant tumor specimens with no prior therapeutic regimens.

**Results:**

Compared with *HER2*-amplified breast and gastric cancers, patients with *HER2*-amplified LUAD showed higher immunogenicity, mainly manifested in immune checkpoints expression and tissue/blood TMB. Additionally, *HER2*-amplified LUAD exhibited an inflamed TIME with remarkably increased genes encoding HLAs, T-cell activity and immune cell-type, and accompanied with tumor‐infiltrating lymphocytes. In LUAD, patients with *HER2* amplification possessed higher tissue TMB than *HER2* mutation, whereas no difference was observed in PD-L1 expression. *HER2* amplification (primary) was associated with significantly higher PD-L1 expression and TMB than acquired *HER2* amplification after resistance to EGFR-TKIs.

**Conclusion:**

Patients with *HER2*-amplified LUAD have better immunogenicity and/or an inflamed TIME among *HER2*-aberrant tumors. Our study may provide clues for establishing the benefits and uses of ICIs for patients with this disease.

## Introduction

Human epidermal growth factor receptor 2 (*ERBB2*, encoding *HER2*) is an oncogenic driver that acts as an “orphan” due to the lack of any known EGF family ligand, which is poised to heterodimerize with other *ERBB* family members to mediate cell proliferation through the Ras-Raf-MAPK and PI3K/Akt signaling pathways (1). *HER2* amplification and mutations are two distinct gene alterations that are also observed in tumors other than breast and gastric cancers, such as LUAD and colorectal cancer (2). *HER2*, also a well-established therapeutic target, is amplified/overexpressed in 12%–20% of breast cancers and 7%–25% of gastroesophageal cancers, for which trastuzumab-based anti-*HER2* therapy has transformed the standard of care with a demonstrated survival benefit (3). *HER2* amplification and mutations are also found in approximately 2-10% of non-small cell lung cancer (NSCLC). *HER2* mutation is recognized as an oncogenic driver in LUAD but the role of *HER2*-amplified is doubtful (4, 5). *HER2* has been shown to participate in the pathophysiology of LUAD, implicating its role as an actionable driver in lung cancers and correlating with poor prognosis (6). In addition, acquired amplification of *HER2* has been proposed as a mechanism of resistance to EGFR/ALK-TKIs, which further confirms its role in tumorigenesis (7). In clinical trials to date, neither trastuzumab nor EGFR/HER2-TKIs have produced clinical benefits in *HER2*-positive NSCLC. Although several novel compounds, such as trastuzumab deruxtecan (T-DXd, DS-8201) and poziotinib, have emerged in recent years, increasing the objective response rate (ORR) to approximately 50% for patients with *HER2*-mutant NSCLC (8, 9), there is currently no approved targeted therapy for *HER2*-amplified LUAD. In view of lacking effective treatment options, there is an urgent need to explore new treatment strategies.

Immune checkpoint inhibitors (ICIs) reinvigorate antitumor immune responses by targeting PD-1/PD-L1 pathways and show remarkable clinical efficacy in driver oncogene-negative NSCLC patients. However, it is generally believed that the response is considerably less frequent in oncogene-addicted NSCLC patients, particularly in patients harboring *EGFR/ALK* variations (10–12). In the first line or above, ICIs among *EGFR*-mutated NSCLC patients showed almost no response and outcomes were far inferior to those of negative driver oncogenes patients (median overall survival 9.8 vs. 16.3 months). However, heterogeneity in response to immunotherapy may exist across different oncogenes in NSCLC. Data from a limited number of patients have shown that ICIs are feasible as monotherapy or combination among *HER2*-mutant lung cancer, with an ORR of 16%-52% and a median progression-free survival (PFS) of 4-6 months (13). However, the place taken ICIs against advanced NSCLC harboring *HER2* amplification remains undetermined. Available evidence shows controversial results in the introduction of ICIs in different *HER-2* amplified tumors. ICIs have generated robust clinical benefits in advanced *HER2*-amplified gastric cancer, whereas there is no significant benefit in *HER2*-amplified breast cancer (14, 15). A higher degree of heterogeneity among tumor types and genomic alteration status obscures our insight into whether *HER2*-aberrant NSCLC and which *HER2* genomic variations would benefit from immunotherapy.

Accumulating evidence suggests that PD-L1 expression, TMB, mismatch repair deficient (dMMR)/microsatellite instability (MSI), immune-related gene expression profiles (GEPs), and tumor‐infiltrating lymphocytes (TILs) represent immunogenicity and TIME features, and correlate with the response to ICIs (16–18). Here, to identify whether immunotherapy has a role in *HER2*-amplified LUAD, we explored and evaluated immunogenicity and TIME traits among breast, lung and gastric tumors with *HER2* amplification. We conducted an integrative analysis that incorporated PD-L1 expression, TMB, MSI status, immune-related and immune cell-type GEPs and measures of TILs from cohorts of TCGA database as well as internal data and patient tumor specimens from 3 hospitals.

## Materials and methods

### Patients selection

Publicly available simple nucleotide variation (SNP), copy number variation (CNV) and transcriptome profiling data from 3 different cohorts were directly downloaded from The Cancer Genome Atlas (TCGA, https://portal.gdc.cancer.gov/repository) dataset: breast invasive carcinoma (BRCA, n =1111), lung adenocarcinoma (LUAD, n = 554), stomach adenocarcinoma (STAD, n = 442).

As a validation set, we collected 298 patients from more than 60 medical institutions in China whose tumor specimens and/or peripheral blood underwent next-generation sequencing (NGS) from February 2019 to February 2021. Comprehensive genomic profiling was screened according to the following criteria: a) known or confirmed pathologic diagnosis of LUAD, STAD and BRCA. b) identified as tier I variants with strong clinical significance. c) exclusion of germline mutations by pairing peripheral blood samples. Ultimately, 288 patients with LUAD, STAD and BRCA were enrolled in our cohort. Additionally, 61% (176/288) of patients’ tumor tissue was tested for PD-L1 expression by Dako 22C3/Ventana SP142. Detailed information including demographic and clinical information is provided in **Table 1**.

**Table 1:** Characteristics of patients in the validation set.

Characteristics	BRCA n=58	STAD n=55	LUAD^1^ n=58	LUAD^2^ n=54	LUAD^3^ n=63
Age, median (range)	52 (26-79)	61 (32-80)	61 (45-91)	58 (29-87)	61 (32-85)
Gender					
Female	58 (100)	13 (24)	18 (31)	25 (46)	40 (63)
Male	0 (0)	42 (76)	40 (69)	29 (54)	23 (37)
Smoking history					
Current or former	8 (14)	29 (53)	35 (60)	17 (31)	15 (24)
Never	26 (45)	20 (36)	13 (23)	21 (39)	35 (55)
Unknown	18 (31)	6 (11)	10 (17)	16 (30)	13 (21)
Treatment history					
Chemotherapy	9 (15)	10 (18)	3 (5)	0 (0)	0 (0)
Targeted therapy	1 (2)	0 (0)	0 (0)	5 (9)	56 (89)
Chemo+ Target	19 (33)	8 (14)	0 (0)	1 (2)	7 (11)
Immunotherapy	0 (0)	1 (2)	0 (0)	1 (2)	0 (0)
Not-received	21 (36)	29 (53)	45 (78)	36 (67)	0 (0)
Unknown	8 (14)	7 (13)	10 (17)	11 (20)	0 (0)
Specimen Type					
Tissue Blood	56 (97) 2 (3)	46 (84) 9 (16)	50 (86) 8 (14)	51 (94) 3 (6)	40 (63) 23 (37)
Sequencing Platform					
HiSeq X Ten	40 (69)	47 (85)	35 (60)	23 (42)^4^	44 (70)
NovaSeq6000	18 (31)	8 (15)	23 (40)	19 (35)	19 (30)
HER2 status					
CNA	58 (100)	55 (100)	58 (100)	–	63 (100)
20 exon insertion	–	–	–	42 (87)^5^	–
Co-*EGFR* mut	–	–	–	–	62 (98)^6^
PD-L1 IHC	51 (88)	35 (64)	35 (60)	28 (52)	27 (43)
TMB detection	51 (88)	45 (82)	33 (57)	30 (56)	24 (38)
MSI detection	51 (88)	45 (82)	38 (67)	28 (52)	27 (43)
MSI-H	0 (0)	0 (0)	0 (0)	0 (0)	0 (0)
HLA-locus^7^	51	51	32	13	23
Heterozygosity	38 (75)	39 (76)	26 (81)	6 (46)	18 (78)
Homozygous	13 (25)	12 (24)	6 (19)	7 (54)	5 (22)

All values are n (%), unless otherwise specified.

BRCA, breast invasive carcinoma; STAD, stomach adenocarcinoma; LUAD, lung adenocarcinoma; CNA, copy number amplification; mut, mutation.

^1^ *HER2* amplification.

^2^ *HER2* mutation.

^3^ *HER2*-acquired amplification.

^4^12 patients detected *HER2* mutation status by ADx-Amplification Refractory Mutation System (ADx-ARMS).

^5^12 patients harboring *HER2* p.S310F.

^6^62 patients received anti-EGFR TKIs; 1 patient received anti-ALK TKIs.

^7^HLA I-heterozygosity, all of the three HLA-I loci (A, B, or C) were heterozygous; HLA I-homozygosity, at least one HLA-I locus (A, B, or C) was homozygous. Patients with HLA I-heterozygous were associated with better survival than HLA I-homozygous when receiving immunotherapy.

To assess PD-L1 expression and immune cell infiltration, we obtained 50 eligible patients’ paraffin blocks from three medical institutions (The First Affiliated Hospital of Xi’an Jiaotong University, Tangdu Hospital and Shaanxi Provincial People’s Hospital), according to the following criteria: a) primary tumors, excluding metastatic sites, b) not received chemotherapy/radiotherapy or other prior to diagnoses, and c) all paraffin blocks were available from January 2020 to May 2021. The clinicopathologic characteristics of the patients are shown in **Table 2**.

**Table 2:** Characteristics of patients enrolled for IHC analysis.

	BRCA	STAD	LUAD^1^	LUAD^2^
Characteristics	n=10	n=10	n=12	n=18
Age, median (range)	58 (44-70)	63 (39-78)	57 (44-76)	53 (31-71)
Sex				
Female	10 (100)	5 (50)	2 (17)	5 (28)
Male	0 (0)	5 (50)	8 (83)	13 (72)
Smoking history				
Current or former	1 (10)	5 (50)	7 (58)	8 (44)
Never	7 (70)	2 (20)	3 (25)	8 (44)
Unknown	2 (20)	3 (30)	2 (17)	2 (11)
ECOG performance status				
0-1	10 (100)	8 (80)	9 (75)	14 (78)
2-4	0 (0)	2 (20)	3 (25)	4 (22)
Stage (TNM)				
I	4 (40)	0 (0)	1 (8)	4 (22)
II	3 (30)	1 (10)	2 (17)	5 (28)
III	2 (20)	5 (50)	3 (25)	2 (11)
IV	1 (10)	4 (40)	6 (50)	7 (39)
Adjuvant therapy				
None	1 (10)	0 (0)	1 (8)	5 (28)
Chemotherapy	0 (0)	6 (60)	3 (25)	6 (33)
Chemo + Target	8 (80)	0 (40)	0 (0)	0 (0)
First line therapy				
Immunotherapy	0 (0)	1 (10)	1 (8)	1 (6)
Chemo + Target	1 (10)	2 (20)	4 (33)	4 (22)
Others	0 (0)	1 (10)	3 (25)	2 (11)
HER2 (IHC)				
0	0 (0)	0 (0)	0 (0)	5 (28)
1+	0 (0)	0 (0)	3 (25)	7 (39)
2+	4 (40)	4 (40)	4 (33)	4 (22)
3+	6 (60)	6 (60)	5 (42)	2 (11)
HER2 status				
FISH ratio≥ 2	10 (100)	10 (100)	–	–
CN≥ 3	–	–	12 (100)	2 (11)
20 exon insertions	–	–	0 (0)	18 (100)

All values are n (%) unless otherwise specified.

BRCA, breast invasive carcinoma; STAD, stomach adenocarcinoma; LUAD, lung adenocarcinoma; ECOG, Eastern Cooperative Oncology Group; Chemo, chemotherapy.

^1^ *HER2* amplification.

^2^ *HER2* mutation.

### Sequencing data analysis

For the TCGA cohort, the masked somatic mutation data were analyzed and visualized using the R package maftools (19). GISTIC 2.0 was used to analyze the downloaded CNV segments. As previously described in detail, A threshold of > 0.2 or < −0.2 is a filtering criterion in the segment mean value for amplification or deletion, respectively (20, 21).

For the validation set, tissue genomic DNA paired with peripheral blood genomic DNA followed the process below: 1) sheared into 150-200 bp fragments; 2) constructed DNA and cell-free DNA libraries; 3) sequenced on an Illumina platform; 4) mapped to the human genome assembly: GRCh37/hg19; 5) filtered with public online genome databases: Exome Aggregation Consortium (ExAC), Genome Aggregation Database (gnomAD) and 1000 Genomes Project (1000G); and 6) identified somatic genome variations. We defined somatic mutation frequency > 1% and gene copy number > 3 as clinically significant (21–23). Additionally, OptiType was used to determine the HLA-I loci (24, 25).

### TMB and MSI analysis

TMB was defined as the total number of somatic nonsynonymous per megabase of tumor tissue which included gene coding errors, base substitution insertions or deletions in detected coding regions. As previously described in detail (26), in the TCGA cohort, TMB was equal to the raw mutation count divided by 38 Mb (the estimate of the exome size). In the validation set, TMB was equal to the number of single-nucleotide variants (somatic nonsynonymous with depth > 100X and allele frequency ≥ 0.05) detected on 1 Mb of the genome (23). As previously described in detail (27), a total of 134 microsatellite loci with a 15-30 bp span were used to calculate the MSI score. Quality control required over 40 loci to pass. The MSI score is equal to the number of unstable loci divided by the number of loci passing quality control, if ≥ 0.3, MSI-H; if < 0.3, MSS/MSI-L.

### Gene expression profiling analyses

In the TCGA cohort, following previous experimental procedures, RNA-sequencing (RNA-seq) data were analyzed using the R package edgeR and normalized by log2-transformation (28). The mRNA expression levels of immune gene signatures, including immune checkpoints, MHC-class-I/II signatures, T cell–inflamed gene expression profiles (GEPs) and immune cell GEPs, were compared among the TCGA-LUAD, TCGA-STAD, and TCGA-BRCA cohorts using the R package limma and were quantified as log2 fragments per kilobase million (FPKM).

The CIBERSORT algorithm, a deconvolution tool with default parameters (29), used a knowledgebase of GEPs and linear support vector regression to estimate the contents of the immune cell expression matrix among the LUAD, STAD and BRCA cohorts.

### IHC

Immunostaining was performed on a Ventana BenchMark XT automated IHC stainer (Roche, Basel, Switzerland) according to the manufacturers’ protocols. The primary antibodies were HER2 (clone 4B5, Roche, Basel, Switzerland); PD-L1 (clone SP263, Roche, Basel, Switzerland); CD4 (clone 2H4A2, Proteintech, Wuhan, China); CD8 (clone C8/144B, Cell Signaling Technology, Danvers, MA, United States); FOXP3 (clone 236A/E7, Abcam, Cambridge, United Kingdom); and CD68 (clone KP1, Maxim-Bio, Fuzhou, China), visualized by the OptiView DAB IHC detection kit (Roche). The stained slides were evaluated separately by two specialized oncologic pathologists blinded to the clinical parameters.

Except for nuclear staining of Foxp3, all markers were positive for membranous staining. The expression of PD-L1 and HER2 was manually assessed according to clinical diagnosis criteria (30, 31). For immune cell analysis, all section images were scanned using a Leica SCN400 slide scanner (Leica Microsystems). The intratumoral regions were evaluated for the density of immune cells (number/mm^2^) under a 20× objective lens field (equal to 0.195 mm^2^) or a 40× objective lens field (equal to 0.0495 mm^2^). Combined with cell morphology and staining intensity, the number of positive immune cells was counted at three to five hot spots and calculated as the average (32, 33).

### Statistical analysis

Data analysis was conducted using R software (version 4.04) and GraphPad Prism (version 9.0.0). Parametric (Student’s t-test and one-way ANOVA) or nonparametric (Mann-Whitney U test and Kruskal-Wallis test) tests were applied depending on whether the data followed Gaussian distribution. Categorical variables were analyzed by the Chi-square test or Fisher exact test. A two-tailed *P <*0.05 was considered statistically significant unless otherwise specified. Additionally, the ggplot2 R package was applied to visualize the boxplot and violinplot, the ComplexHeatmap R package was used to visualize the Heatmap and the fmsb R package was used to visualize the Radar Chart.

## Results

### Correlation between *HER2* amplification and PD-L1/other immune checkpoints expression in LUAD patients compare to BRCA and STAD

**Figure 1:**
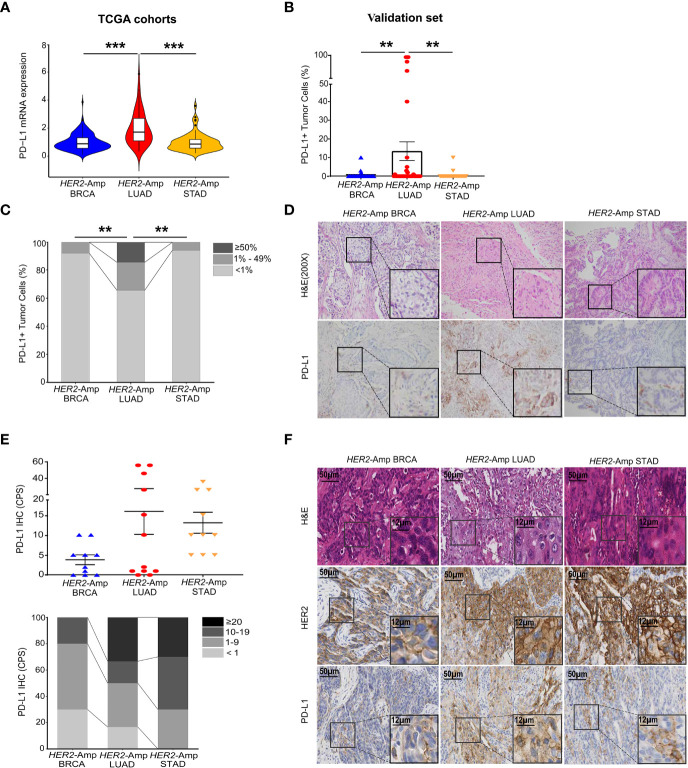
Correlation between *HER2* amplification and PD-L1 and/or other immune checkpoint expressions in patients with BRCA, LUAD and STAD. **(A)** PD-L1 mRNA expression among the *HER2-*amplified BRCA cohort (n = 212), *HER2*-amplified LUAD cohort (n = 56) and *HER2-*amplified STAD cohort (n = 71) based on analysis of the TCGA database. **(B, C)** Statistical results of PD-L1 staining in *HER2*-amplified cohorts of 51 BRCA patients, 35 LUAD patients and 35 STAD patients. PD-L1 expression in tumor cells is classified as ≥ 50%, 1% to 49% and < 1%. **(D)** Representative images of hematoxylin-eosin (HE) staining and PD-L1 IHC staining in BRCA, LUAD and STAD tissues with *HER2* amplification. **(E, F)** Statistical analysis **(E)** and images **(F)** of HE, HER2 and PD-L1 IHC staining were conducted. PD-L1 expression was assessed at cut-offs of ≥ 20, 10 to 19, 1 to 9 and < 1 based on the combined positive score (CPS). CPS = [(number of PD-L1-positive tumor cells and mononuclear inflammatory cells)/(total number of tumor cells)] × 100. Amp, amplified; BRCA, breast invasive carcinoma; LUAD, lung adenocarcinoma; STAD, stomach adenocarcinoma. *P* < 0.05 was regarded as significantly different. ***P < 0.001, **P < 0.01.

PD-L1 expression has high predictive value in guiding cancer immunotherapy (17). Thus, we first investigated PD-L1 expression levels between *HER2* amplification and PD-L1 expression in BRCA, LUAD and STAD cohorts by interrogating and analyzing RNA-seq data from TCGA. The level of PD-L1 mRNA expression in *HER2*-amplified LUAD was significantly higher than that in *HER2*-amplified STAD and BRCA cohorts (**Figure 1A**). However, no differences were noted between *HER2*-amplified STAD and BRCA cohorts. Further analysis confirmed this result at the PD-L1 protein level (**Figures 1B–D**).

Except for tumor cells, PD-L1 is also expressed in immune cells. To eliminate the singleness of the PD-L1 evaluation method and further confirm the association between *HER2* amplification and PD-L1 expression as the TCGA cohorts and validation set demonstrated, we detected 10 BRCA, 12 LUAD, 10 STAD surgical or biopsy specimens using IHC. Immunostaining showed that *HER2*-amplified LUAD specimens tended to have stronger staining for the PD-L1 protein assessed by the combined positive score (CPS, **Figure 1E**). In addition, STAD harboring *HER2* amplification equally expressed higher PD-L1 owing to its immune cell staining within the tumor stroma. However, *HER2*-amplified BRCA continued to lower the expression of PD-L1 when assessing both tumor and immune cells (**Figure 1F**).

To figure out the association between *HER2* amplification and other non–PD-L1 immune checkpoints in BRCA, LUAD and STAD patients, we next exploit RNA-seq data to depict the expression levels of 7 key immune checkpoints. A heatmap displayed remarkably increased expression of checkpoints in the *HER2*-amplified LUAD cohorts while decreased expression in the *HER2*-amplified BRCA and STAD cohorts. More interestingly, PD-L1 mRNA expression was markedly increased in the *HER2*-amplified LUAD subgroup, relative to other immune inhibitory checkpoints. Finally, boxplots were constructed to represent the other two most significantly different immune checkpoints (PD-1 and IDO1; **Figures S1A, S1B**).

### Correlation between *HER2* amplification and TMB in LUAD patients compare to BRCA and STAD

**Figure 2:**
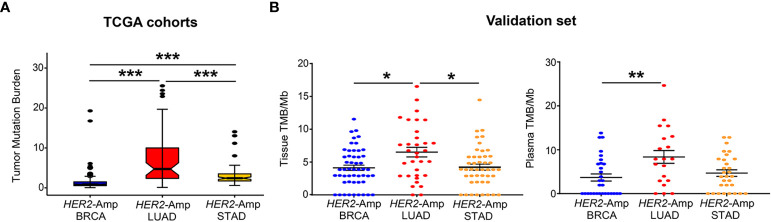
Correlation between *HER2* amplification and TMB in patients with BRCA, LUAD and STAD. **(A)** Tissue TMB driven by *HER2* amplification among three tumors from TCGA cohorts. **(B)** Tissue and blood TMB driven by *HER2* amplification among three tumors from the validation set. TMB, tumor mutation burden, was defined as the total number of somatic nonsynonymous per megabyte. *P* < 0.05 was regarded as significantly different. ***P < 0.001, **P < 0.01, *P < 0.05.

TMB is an independent predictor/indicator of response to immunotherapy in pan-cancers (34). From TCGA cohort analysis, patients with *HER2*-amplified LUAD showed significantly higher tissue TMB than *HER2*-amplified BRCA and STAD cohorts (**Figure 2A**). Consistent with the finding from the discovery set, our validation set manifested a similar result: *HER2*- amplified LUAD patients had higher tissue TMB and/or blood TMB than the other two cohorts, particularly *HER2*-amplified BRCA cohorts (**Figure 2B**).

### The landscape of the TIME among *HER2*-amplified BRAC, LUAD and STAD

**Figure 3:**
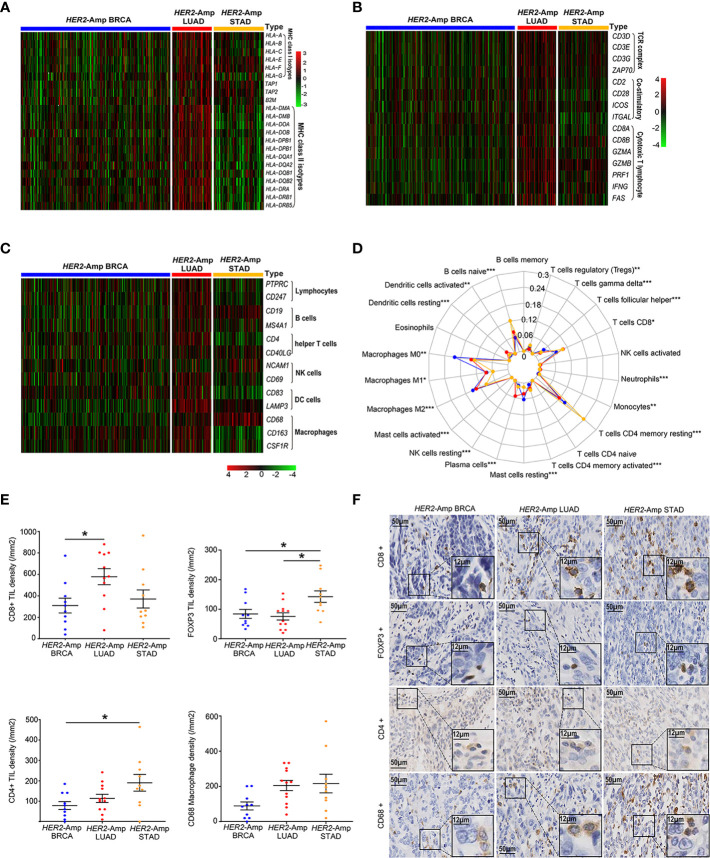
The landscape of the TIME among *HER2*-amplified BRAC, LUAD and STAD. **(A)** Heatmap depicting the expression of HLA-related gene profiles among three distinct tumors from RNA-seq. **(B)** Heatmap depicting the process of T cell activation, including antigen recognition, signal transduction and T cell immune efficacy from RNA-seq data. **(C)** Heatmap depicting the mRNA expression levels of the immune cell-associated gene signature. **(D)** A radar chart displaying the fraction of infiltrated immune cells by CIBERSORT. The blue, red and yellow lines represent *HER2*-amplified BRAC, LUAD and STAD, respectively. **(E)** Comparison of TIL and macrophage densities at intratumoral regions among patients with *HER2*-amplified LUAD (n=12), *HER2*-amplified BRCA (n=10) and *HER2*-amplified STAD (n=10). Note: The paraffin section of 1 patient with *HER2*-amplified LUAD failed the quality control after CD8 IHC staining and was not included in the statistical analysis. **(F)** Representative images of HE staining and IHC staining of HER2, CD4, CD8, FOXP3 and CD68 among the above-mentioned tumors. *P* < 0.05 was regarded as significantly different. ***P < 0.001, **P < 0.01, *P < 0.05.

Anti-tumor immunity requires antigen presentation, and T cell priming and trafficking to the tumor tissue. These steps require the coordinated activity of immune networks within the TIME. We used TCGA-RNAseq data and applied the IHC method to depict the whole process as comprehensively as possible. Compared with the other two cohorts, genes encoding classical MHC class I/II antigens and related antigen processing machinery proteins (TAP1 and B2M) were expressed at significantly higher levels in *HER2*-amplified LUAD cohorts (**Figure 3A**). Additionally, *HER2*-amplified LUAD possessed increased immune-related GEPs (**Figure 3B**), such as T-cell antigen receptor (CD3), costimulatory molecules (CD28, ICOS, etc.) and cytotoxic effect-related genes (CD8A, IFNG, GZMA, etc.). To sufficiently assess the contents of immune cell infiltration, CIBERSORT algorithm and immune cell-related genes were used to quantify various immune populations (**Figures 3C, D**). The absolute number of multiple immune cell populations, including CD4^+^, CD8^+^ T cells and M1 macrophages, were markedly increased in *HER2*-amplified LUAD. Additionally, regulatory T cells (Tregs), a type of suppressive T cell, were lower infiltration in *HER2*-amplified LUAD cohorts.

To further make our preliminary results more convincing, we conducted IHC to evaluate the density of immune cell infiltration among patients with *HER2*-amplified BRCA, LUAD and STAD. As expected, we observed increased CD8^+^ TIL densities and decreased FOXP3^+^ TIL densities in *HER2* amplified LUAD specimens compared to *HER2*-amplified BRCA and STAD specimens. In addition, we found increased CD4^+^ TILs densities in *HER2*-amplified STAD specimens, corresponding to upregulation of FOXP3^+^ Tregs. The densities of CD68 macrophages were similar between the *HER2*-amplified STAD and LUAD groups (**Figures 3E, F**). Regardless of the subtype of immune cells, *HER2*-amplified BRCA exhibited lower TIL and macrophage densities than the other two groups. Generally, the IHC results are consistent with the RNA-seq analysis.

### The immunogenicity and TME in *HER2*-aberrant LUAD

**Figure 4:**
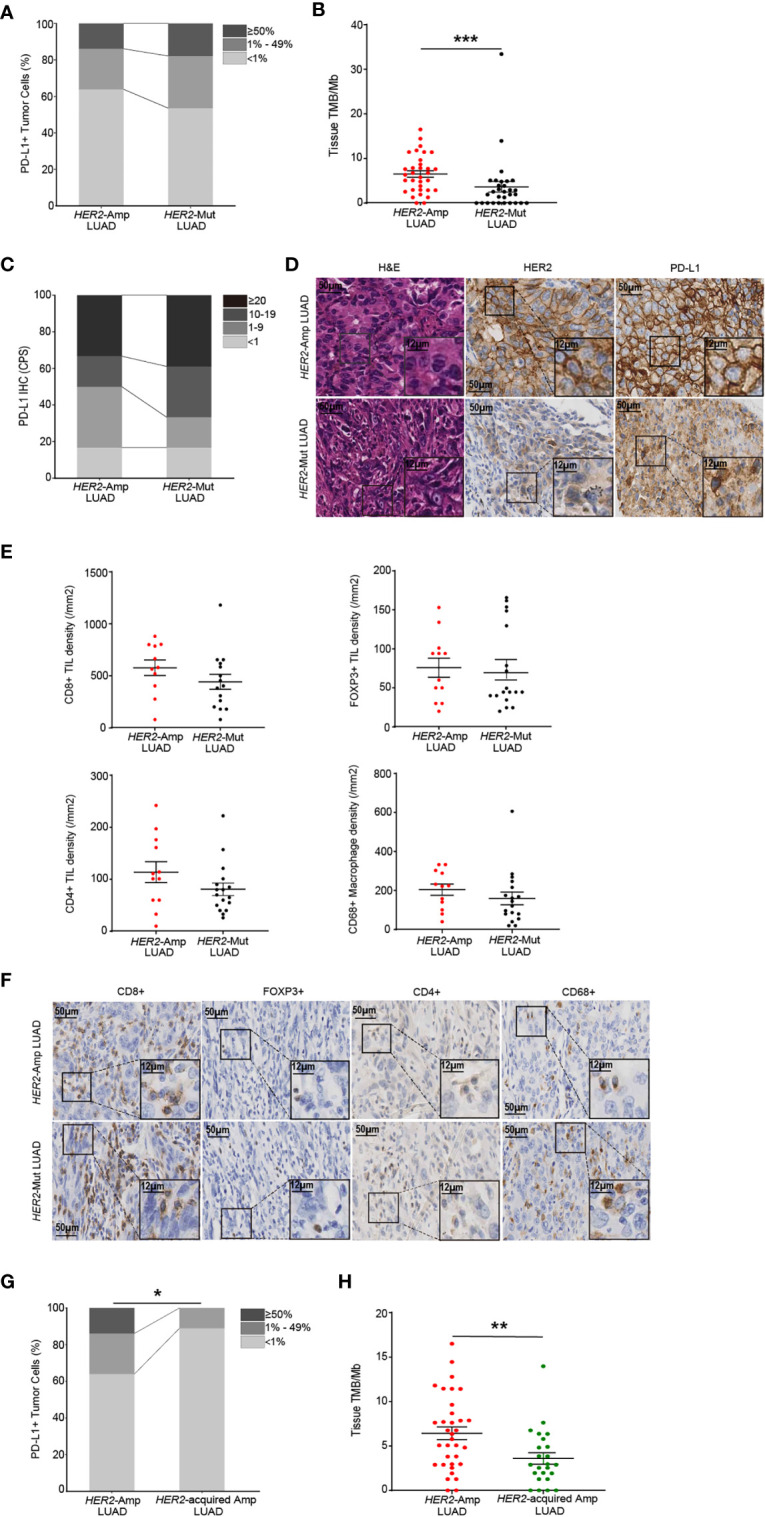
The immunogenicity and TIME in *HER2*-aberrant LUAD. **(A, B)** Difference in PD-L1 expression on tumor cells **(A)** and the level of TMB **(B)** between amplification and mutation. **(C, D)** Statistical results (c) and images **(D)** of PD-L1 expression evaluated by CPS. **(E, F)** Densities of CD8^+^, POXP3^+^ and CD4^+^ lymphocytes, and CD68^+^ macrophages **(E)** between LUAD patients with *HER2* amplification (n=12) and mutation (n=18). Corresponding IHC images are shown in **(F)**. Note: The paraffin section of 3 patients with *HER2*-mutant LUAD failed the quality control after CD8 IHC staining and were not included in the statistical analysis. 1 patient with *HER2*-mutant LUAD was not included after CD4 IHC staining because of the same problem. **(G, H)** The PD-L1 **(G)** and TMB **(H)** levels in LUAD patients with *HER2* amplification and acquired *HER2* amplification. *P* < 0.05 was regarded as significantly different. ***P < 0.001, **P < 0.01, *P < 0.05.

Next, we investigated the PD-L1, TMB and immune cell infiltrations between *HER2* amplification and mutation in LUAD. A significantly higher TMB was apparent for patients with *HER2* amplification, but no difference was observed in PD-L1 expression (**Figures 4A–D**). We next evaluated immune cell densities in patients with *HER2* amplification and *HER2* mutation. Although no significant difference was obtained regarding the TIL and macrophage densities, there was a tendency for both CD8^+^ and CD4^+^ TIL counts to be higher in tumors harboring *HER2* amplification than in those harboring *HER2* mutation (**Figures 4E, F**).

In addition, amplification of *HER2* is regarded as one of the resistance mechanisms in NSCLC patients after receiving anti-EGFR/ALK TKIs [7]. We distinguished acquired *HER2* amplification through co-mutation status and treatment history and demonstrated that acquired *HER2* amplification LUAD rendered lower PD-L1 expression and TMB than *HER2* amplification (naïve or primary amplification, **Figures 4G, H**). Together, these findings supported that *HER2* amplification represents a high degree of immunity and immunogenicity among *HER2*-aberrant NSCLC.

## Discussion

The characteristics of the immunogenicity and TIME in *HER2*-amplified LUAD have not yet been illustrated. The lower incidence of *HER2* amplification or mutation in LUAD leaves challenges in depicting the landscape of immunogenicity and TIME features when compared with wild-type NSCLC patients. Thus, we explored the correlation between *HER2* amplification and characteristics of immunogenicity and TIME in LUAD contrast to gastric cancer that benefited from immunotherapy (15), and breast cancer that did not (17), to establish a theoretical feasibility of ICIs for *HER2*-amplified LUAD. Here, we found that patients with *HER2*-amplified LUAD showed higher immunogenicity, mainly manifested in PD-L1 expression at the mRNA and protein level, as well as tissue and blood TMB, in comparison with *HER2*-amplified breast and gastric cancers. Additionally, *HER2*-amplified LUAD exhibited an inflamed TIME with remarkably increased genes encoding HLAs, T-cell activation and immune cell-type, and accompanied with TILs. In LUAD, we observed that patients with *HER2* amplification possessed elevated TMB than those with *HER2* mutation, whereas no difference was observed regarding PD-L1 expression. *HER2* amplification (naïve or primary amplification) was associated with significantly increased PD-L1 expression and higher TMB than acquired *HER2* amplification after resistance to *EGFR*-TKIs. The pooled results support that *HER2*-amplified LUAD captures a higher likelihood of deriving benefits from ICIs.

It is well known that NSCLC as a whole has higher PD-L1 expression and TMB relative to breast and gastric cancers, but the whole character could not accurately define the individuals, as *EGFR*-mutant NSCLC and triple-negative breast cancer (TNBC), which represented the different immunogenicity and generated an opposite immune response to ICIs (10, 35). It is believed that specific oncogenes represent their own unique immunogenicity and TIME within the tumor. As per available literature in NSCLC, higher PD-L1 expression and TMB are frequently observed in male smokers and in patients harboring *KRAS* mutation, whereas there is no correlation with *EGFR* alterations (12, 36). We found that PD-L1 expression at the mRNA and protein level, as well as tissue and blood TMB are dominant in *HER2*-amplified LUAD compared with breast and gastric cancers harboring *HER2* amplification. In essence, it can be seen from the literature that *HER2*-amplified LUAD was associated with male smokers and invasive features (37). An RTDs study confirmed that *HER2* amplified NSCLC tumors were associated with higher TMB (100% of tumors≥10 muts/Mb), although the study only involved 6 patients (38). In comparison, prior studies indicated that increased PD-L1 expression and TMB in breast cancer were enriched in TNBC and lobular carcinomas (39). In gastric cancers, *HER2* amplification is inversely correlated with PD-L1 expression (40). Bioinformatics research indicated that *HER2* amplification was not classified into PD-L1 amplification and hypermutation subtypes of gastric cancer (41). Such findings could be the reason *HER2*-amplified LUAD shows increased immunogenicity compared with breast and gastric cancer.

Little information is available regarding the correlation between the TIME and *HER2* amplification in NSCLC. Conventional opinion is that breast cancers bearing *HER2* amplification result in a non-inflamed TIME, with relatively low infiltration of intratumor TILs (39, 42). In contrast, *HER2*-amplified gastric cancer was dominant in the gastroesophageal junction (GEJ) and intestinal histology, connected with microbial infection, which generates a chronic inflammation status (41, 43). In patients with *HER2*-amplified LUAD, exposure to accumulated mutation load can release neoantigens that trigger T cell activation and recruit immune cell infiltration. Hence, as we demonstrated, *HER2*-amplified LUAD samples showed increased immune-related and immune cell-type gene expression profiles (GEPs) compared with the other two cancer groups, especially the *HER2*-amplified BRCA group. The presence of a greater number of TILs and fewer Tregs has been observed in *HER2*-amplified LUAD regardless of RNA-seq and IHC results. Of note, the upregulation of CD4^+^ TILs is accompanied by FOXP3^+^ Treg infiltrations in *HER2*-amplified gastric cancer. In general, in comparison with breast and gastric tumors harboring *HER2* amplification, these pooled results implicated the possibility that *HER2*-amplified LUAD establishes a favorable TIME for developing therapeutic efficacy in cancer immunotherapy.

Among *HER2*-aberrant NSCLC, *HER2* mutation mainly occurs in female no-smokers, performing similar clinicopathological characteristics to *EGFR* mutation, and acquired *HER2* amplification is a mechanism of resistance to EGFR-TKIs (7). We observed that patients with *HER2* mutation and acquired *HER2* amplification possessed lower TMB than *HER2* amplification. Prior studies pointed out that the non-inflamed TIME for patients with NSCLC harboring *EGFR* mutation is thought to be reflective of and caused by their low TMB (42), which naturally accounts for our observation. However, unlike available literature suggesting that *HER2*-mutant NSCLC has been shown to have lower PD-L1 expression than wild-type NSCLC (44), we discovered that cases with *HER2* mutation exhibited higher PD-L1 expression at the protein level, corresponding to dense TILs. This phenomenon is similar to the conflicting results regarding the relationship between PD-L1 and *EGFR* mutation (45). In addition, we found that patients with acquired *HER2* amplification had lower PD-L1 expression than those with primary amplification. Although a recent study indicated that EGFR-TKI treatment was associated with a significant increase in PD-L1 expression in *EGFR*-mutant NSCLC, the impact of cytotoxic chemotherapy is not excluded and specific to acquired *HER2* amplification remains underdetermined (33). In brief, *HER2* amplification (naïve or primary amplification) represents a high degree of immunity and immunogenicity among *HER2*-aberrant LUAD.

The lower frequency of *HER2* amplification or mutation in NSCLC imposes restrictions on the analysis of the immunotherapy effect. Our study was necessarily limited in that the efficacy of ICIs targeting NSCLC patients harboring *HER2* alterations is lacking. It is generally believed that tumors bearing an inflamed phenotype are conducive to recognition by the immune system and further improve the clinical benefits of immunotherapy. Existing evidence demonstrated that patients with *HER2*-amplified gastric cancer could benefit from ICIs treatment rather than *HER2*-amplified breast cancer, and our findings suggest that *HER2*-amplified LUAD shows higher immunogenicity and a more “inflamed” TIME than *HER2*-amplified breast cancer and gastric cancer. Thus, we speculate that there are good prospects for practical applications of ICIs in *HER2*-amplified LUAD that can benefit from immunotherapy. However, the consistency between immune markers and the efficacy of immunotherapy in *HER2*-amplified LUAD remains elusive and represents an area of further comprehensive evaluation.

In summary, we conducted an assessment of immune-related biomarkers and elucidated an “inflamed” phenotype of *HER2*-amplified LUAD among *HER2*-positive tumors, which would provide a theoretical basis for the practical application of ICIs and accelerate the pace toward immuno-precision direction.

## Data availability statement

All data generated or analyzed during this study are included in this publish article/**Supplementary Material**. Further inquiries can be directed to the corresponding author.

## Ethics statement

The studies involving human participants were reviewed and approved by the Ethics Committee of The First Affiliated Hospital of Xi’an Jiaotong University (No.XJTU1AF2021LSK-3379). The work was conducted in accordance with the Declaration of Helsinki. 50 patients who obtained eligible paraffin blocks have signed written informed consent in accordance with the ethical guidelines, their clinicopathological information was captured from the Electronic Medical Record (EMR).

## Author contributions

Conceived and designed the analysis: QW, ZM, WL, YJ and HG. Contributed data or analysis tools: SW, LW, LC, ZY, XF, PJ, YB, LX, SZ. Performed the analyses: XJ, LJ, ML and GZ. Wrote the paper: QW, ZM, WL, YH, YJ and HG. All authors contributed to the article and approved the submitted version.
