# Supplementary material for: Characteristics of the immunogenicity and tumor immune microenvironment in HER2-amplified lung adenocarcinoma
Source: Front Immunol. 2022 Dec 15;13:1042072. doi: 10.3389/fimmu.2022.1042072 (PMC9797999; doi:10.3389/fimmu.2022.1042072)
Supplement: Supplementary file 1 [file DataSheet_1.pdf]

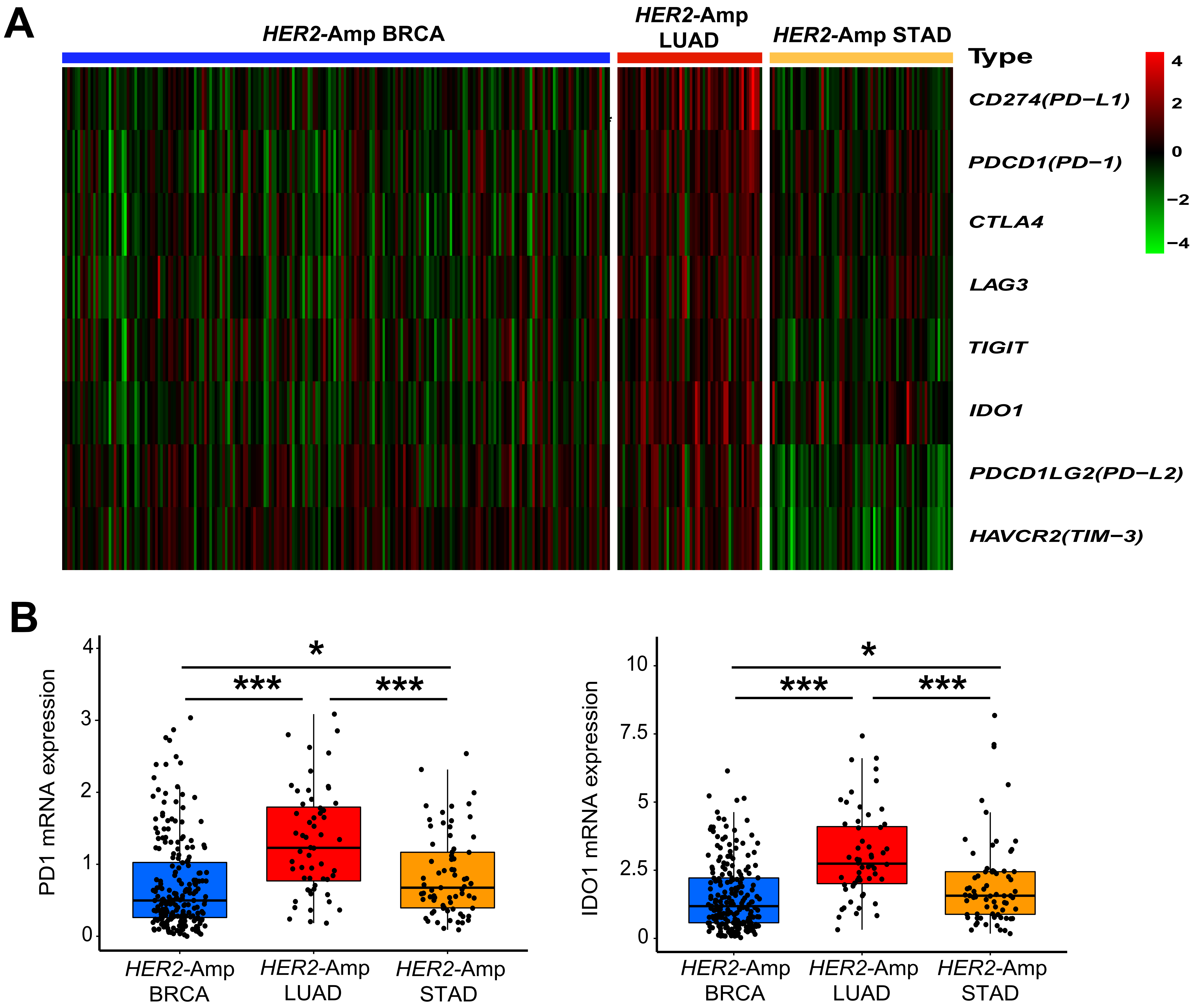

Supplemental Figure 1. The expression of immune-related checkpoints in patients with *HER2* amplification among BRCA, LUAD and STAD. A. Heatmap representation of relative mRNA expression levels of immune-related checkpoints from TCGA-RNAseq data. B. Quantitative analysis of significantly different expression of two immune checkpoints (PD-1 and IDO1) in *HER2*-amplified cohorts. Amp, amplification; BRCA, breast invasive carcinoma; LUAD, lung adenocarcinoma; STAD, stomach adenocarcinoma; $P < 0.05$ was regarded as significantly different. ***$P < 0.001$, **$P < 0.01$, *$P < 0.05$.
